# Contextual Visual Cues in Dessert Evaluation: A Mixed-Effects Analysis of Plate Color Effects on Sweetness and Bitterness Perception

**DOI:** 10.3390/foods15101629

**Published:** 2026-05-07

**Authors:** Marcos Eduardo Valdés-Alarcón, Luis Alfonso Benalcázar-Carvajal, Alexander Sánchez-Rodríguez, Rodobaldo Martínez-Vivar, Gelmar García-Vidal, Reyner Pérez-Campdesuñer

**Affiliations:** 1 Faculty of Gastronomic Sciences and Tourism, Universidad UTE, Quito 170508, Ecuador; marcos.valdes@ute.edu.ec (M.E.V.-A.); luis.benalcazar@ute.edu.ec (L.A.B.-C.); 2 Faculty of Engineering Sciences and Industries, Universidad UTE, Quito 170527, Ecuador; 3 Faculty of Law, Administrative and Social Sciences, Universidad UTE, Quito 170527, Ecuador; rodobaldo.martinez@ute.edu.ec (R.M.-V.); gelmar.garcia@ute.edu.ec (G.G.-V.); reyner.perez@ute.edu.ec (R.P.-C.)

**Keywords:** sensory evaluation, crossmodal correspondences, plate color, mixed-effects models, repeated measures, consumer perception

## Abstract

Flavor perception is a multisensory process shaped not only by foods’ physicochemical properties but also by contextual cues that influence consumer expectations. Plate color is one such cue, yet evidence remains limited for desserts rooted in Latin American culinary traditions. This study examined whether plate color (red vs. black) alters perceived sweetness and bitterness in cacao- and passion fruit-based desserts. Using a randomized within-subject crossover design, adult consumers evaluated products served on both red and black plates and rated sweetness and bitterness on 100-point visual analog scales. Sourness, overall liking, and consumption intention were also collected for complementary purposes but were not included as primary outcomes in this analysis. Data were analyzed using linear mixed-effects models to account for repeated measures and individual-level variability. Results revealed a significant plate color × product interaction: red plates increased perceived sweetness in the passion fruit-based dessert, whereas black plates intensified perceived bitterness in the cacao-based dessert. Food neophobia did not significantly moderate these effects. Overall, the findings demonstrate that plate color systematically modulates reported taste intensity in a product-dependent manner. These results extend evidence on color–taste crossmodal correspondences and provide practical insights for sensory-driven food presentation and product design.

## 1. Introduction

Flavor perception is widely recognized as a complex multisensory phenomenon arising from the integration of gustatory, olfactory, tactile, and visual inputs, rather than being determined solely by the intrinsic physicochemical properties of foods. Over the past two decades, research in sensory and consumer science has increasingly demonstrated that contextual cues surrounding food consumption can systematically bias both perceived sensory intensity and hedonic evaluation, thereby shaping eating experiences and downstream behavioral responses [1,2,3]. Within this framework, visual information plays a particularly prominent role, as it is typically processed prior to tasting and serves as a powerful source of expectations that influence subsequent sensory judgments.

Among visual contextual cues, plate color has emerged as a salient factor that modulates flavor perception by activating learned associations and anticipatory beliefs before food is consumed. A growing body of empirical evidence indicates that dark colors, such as black and brown, are often associated with bitterness and greater sensory intensity, whereas red and pink hues are more frequently associated with sweetness, even when the food itself remains unchanged [4,5,6,7]. These effects are commonly interpreted through the theoretical lens of crossmodal correspondences, which refers to systematic, non-arbitrary associations between attributes across different sensory modalities. Across cultures and product categories, robust correspondences have been documented between red hues and sweetness, yellow-green tones and sourness, and dark colors such as black or brown and bitterness [8,9,10,11]. Importantly, such correspondences not only shape expectations but also directly influence perceived taste intensity and affective responses during consumption [12].

From a cognitive perspective, the influence of plate color on flavor perception can be further explained by expectation-based mechanisms, particularly the assimilation–contrast framework. According to this framework, visual cues generate anticipatory expectations that bias sensory perception once the food is tasted. When the expectation induced by a visual cue is congruent with the intrinsic sensory properties of the product, perceptual judgments tend to assimilate toward the expectation, resulting in enhanced intensity or liking. Conversely, when expectations are incongruent, contrast effects may occur, leading to attenuation or exaggeration of perceived sensory attributes [13,14,15]. Evidence from recent experimental studies suggests that both assimilation and contrast processes may operate depending on the salience of the visual cue, the dominance of specific taste attributes, and the product category under evaluation, underscoring the need for carefully controlled experimental designs in order to disentangle expectation-driven effects from other contextual sources of variation [13,14,15,16].

Methodologically, isolating plate color effects from other contextual influences remains challenging, particularly in consumer settings where multiple extrinsic cues may operate simultaneously [13,16]. Many previous studies have relied on between-subject designs, which may lack sensitivity to detect subtle perceptual effects due to interindividual variability [17,18]. In contrast, within-subject crossover designs allow each participant to serve as their own control, thereby improving statistical efficiency in repeated sensory evaluations. When combined with linear mixed-effects models, this approach enables more robust estimation of treatment effects by accounting for intra-individual correlation and individual differences [18,19]. Accordingly, randomized and counterbalanced crossover designs are increasingly recommended for the study of visual–gustatory interactions. The present study, therefore, employs a controlled crossover design analyzed using mixed-effects modeling to examine contextual sensory effects.

Despite the growing body of empirical evidence on color–taste crossmodal correspondences and the increasing use of controlled experimental designs in sensory research, several limitations remain, particularly regarding ecological validity and the interaction between visual cues and culturally embedded food contexts. First, empirical evidence on plate color effects has been disproportionately generated in European, North American, and East Asian contexts, raising questions about the extent to which current theoretical models generalize across food cultures. Second, relatively little attention has been devoted to products with strong cultural or patrimonial significance, even though such foods often possess complex sensory profiles that may be particularly sensitive to contextual modulation [20,21]. Addressing these gaps is essential not only for improving the external validity of crossmodal correspondence research but also for understanding how visual context operates in diverse culinary traditions.

Cacao- and passion fruit-based desserts provide a theoretically relevant context for examining these issues because they combine cultural familiarity with clearly differentiated dominant sensory profiles. In crossmodal research, products with contrasting intrinsic taste characteristics are especially useful for assessing whether visual cues interact with the food’s most salient sensory attribute. In this regard, cacao-based products are typically characterized by a balance between bitterness, sweetness, and cacao intensity, with consumer acceptance strongly influenced by how these attributes are managed. Prior research indicates that excessive bitterness and acidity are negatively associated with liking, whereas perceived sweetness and cacao flavor intensity are positively related to preference [22]. Passion fruit products, by contrast, are generally defined by pronounced acidity, intense aroma, and a distinctive sweet–sour balance, making them particularly relevant for examining how visual cues may shape anticipatory judgments before tasting [23,24].

Moreover, in both cacao- and passion fruit-based products, existing studies have focused primarily on formulation, processing, and acceptance variables, leaving the role of visual presentation—such as plate color—largely unexplored [22,23,24]. From this perspective, the present study uses traditional desserts not as a matter of convenience but as ecologically relevant stimuli to test how plate color interacts with intrinsic sensory profiles in familiar culinary contexts.

More broadly, plate color should be understood as one element within a constellation of contextual cues that jointly shape sensory evaluation. Research has shown that factors such as lighting conditions, tableware material, consumption environment, and serving temperature can influence perceived quality, flavor intensity, and hedonic judgments [13,16]. Because such factors may alter sensory expectations and evaluation, controlled experimental studies should standardize them whenever they are not part of the focal manipulation. In the present study, serving temperature was therefore held constant across treatments in order to isolate the effect of plate color more rigorously. At the same time, the literature reports heterogeneous findings, including weak or null effects in some cases, suggesting that contextual influences are contingent rather than universal and may depend on product characteristics or individual differences, such as food-related personality traits [8].

Taken together, the literature indicates that visual contextual cues can systematically bias flavor perception, but rather the outcome of dynamic multisensory integration in which visual contextual cues play a decisive role. Research on expectation assimilation and color–taste crossmodal correspondences suggests that plate color can systematically bias perceived sweetness and bitterness; however, the magnitude and direction of these effects depend on the food’s sensory profile and the congruence between visual cues and intrinsic product characteristics. Moreover, existing evidence indicates substantial heterogeneity across products and contexts, underscoring the need for well-controlled experimental designs that can disentangle main effects from interaction effects in repeated-measures consumer data. Against this background, the present study investigates how plate color modulates the perception of sweetness and bitterness in cacao- and passion fruit-based products with contrasting sensory profiles, using a rigorous within-subjects crossover design to clarify the conditions under which visual context amplifies or attenuates dominant taste attributes.

Drawing on the literature on crossmodal correspondences and expectation-based modulation of flavor perception, the present study tests the following hypotheses:

**H1.**  *Plate color interacts with product type to produce differences in perceived sweetness and bitterness under controlled serving conditions, without changes to the food’s intrinsic physicochemical properties. This hypothesis therefore focuses on the interaction between visual context and product characteristics, rather than on a uniform main effect of plate color.*

**H2.**  *The effect of plate color depends on the product’s dominant sensory profile. To specify this expectation, two product-specific sub-hypotheses were formulated:*

**H2a.**  *In cacao-based desserts, black plates increase perceived bitterness relative to red plates.*

**H2b.**  *In passion fruit-based desserts, red plates increase perceived sweetness relative to black plates.*

**H3.**  *Individual differences in food-related personality traits (e.g., food neophobia) exert only a limited moderating influence on the relationship between plate color and flavor perception.*

A controlled experimental study was therefore conducted in which participants evaluated cacao- and passion fruit-based desserts served on two plate colors (red and black) using a within-subject crossover design.

## 2. Materials and Methods

The study employed a randomized within-subject crossover experimental design. Each participant evaluated two prepared desserts—one cacao-based dessert and one passion fruit-based dessert—served on plates of two different colors (red and black), yielding four experimental conditions per participant. Participants were not informed about the specific research objective, although the visual manipulation (plate color) remained observable as part of the tasting procedure. Random coding and randomized presentation order were implemented to reduce expectation and order effects. This design allowed each individual to serve as their own control, thereby reducing interindividual variability and increasing statistical power.

The samples evaluated consisted of prepared desserts rather than raw ingredients. Specifically, one sample was a cacao-based dessert, and the other was a passion fruit-based dessert. These products exhibit distinct sensory profiles: cacao-based desserts are typically characterized by bitterness and cacao intensity, modulated by sweetness, whereas passion fruit-based desserts are defined by pronounced acidity, intense fruity aroma, and a dynamic sweet–sour balance. These characteristic sensory profiles provide a relevant basis for interpreting how visual contextual cues may interact with dominant taste attributes.

Sensory evaluations were conducted in a controlled indoor environment under standardized experimental conditions in order to minimize external distractions and sensory interference. The desserts were served on round, flat ceramic plates of identical dimensions, differing only in color (red or black). Each plate measured 22 cm in diameter and had a matte finish. During evaluation, the plates were placed on the same wooden table surface across all conditions. The evaluation sessions were conducted in a room of approximately 70 m^2^, with stable ambient conditions and minimal external distractions.

Lighting was kept constant, with a correlated color temperature of approximately 4000 K and a homogeneous illuminance of approximately 500 lux on the evaluation surface, verified using a lux meter with color temperature measurement. Portion size (30 g), serving temperature (8 ± 1 °C), utensil type (standard metal spoon), and the maximum time between plating and evaluation (≤2 min) were held constant across all conditions. Each dessert was served centrally on the plate to ensure consistent visual exposure to the surrounding plate color. Given the standardized portion size, the dessert occupied only part of the plate surface, leaving a visible border of plate color in all conditions. The servings were identified using random alphanumeric codes to ensure double blinding. Between conditions, participants cleansed their palate using water and unsalted crackers.

Adult consumers (≥18 years of age) were recruited via an open, voluntary invitation disseminated within the university environment prior to the sensory evaluation sessions. Participation was based on self-selection and subsequent eligibility screening according to the predefined inclusion and exclusion criteria. Inclusion criteria required the absence of allergies or intolerances related to cacao or passion fruit, as well as no self-reported taste or olfactory impairments. Participants presenting transient conditions likely to affect sensory perception on the day of evaluation—such as respiratory infections, nasal congestion, general discomfort, recent consumption of alcohol, tobacco, or highly seasoned foods, or the use of medications with potential effects on taste or smell—were excluded. These conditions were verified by self-report prior to the session. Participation was voluntary, written informed consent was obtained from all participants prior to participation, and the study was conducted in accordance with established ethical principles for research involving human subjects.

The target sample size was set at a minimum of 100 participants, following methodological recommendations for sensory studies using crossover designs. This threshold was further supported by a simulation-based power analysis for linear mixed-effects models, assuming small-to-moderate effect sizes and within-subject correlation [19,25]. To account for possible attrition or incomplete data, an additional 10% margin was incorporated, yielding a recruitment target of 110 participants [26].

Accordingly, 110 participants were recruited for the study. Of these, five were excluded prior to analysis due to incomplete evaluations, resulting in a final analyzed sample of 105 participants. All participants included in the final analysis completed all within-subject conditions, evaluating both product types across the four experimental conditions, thus yielding a complete crossover dataset with no condition-level missing observations. The final sample comprised 43 women and 62 men, with a mean age of 41.5 years (SD = 9.3; range = 21–67).

The products were prepared using standardized and previously documented recipes, with ingredients, proportions, preparation procedures, and processing conditions held constant to ensure product homogeneity across all evaluations. The cacao-based dessert consisted primarily of cacao-based components, sugar, milk, and stabilizing ingredients, whereas the passion fruit-based dessert was prepared using passion fruit pulp, sugar, water, and gelling or stabilizing agents. No additional ingredients were varied across conditions. Plates were identical in shape, size, and material, differing only in nominal color (red and black). At the time of serving, the cacao-based dessert had a dark brown appearance, whereas the passion fruit-based dessert presented a yellow-to-orange appearance. Instrumental color measurements in CIELAB coordinates were not recorded for the plates or samples under the original experimental conditions. The selection of plate colors (red and black) was informed by prior research on color–taste crossmodal correspondences.

At the beginning of the session, participants completed a structured questionnaire administered in self-report format. The instrument included items assessing hunger level (measured on a numerical scale), prior familiarity with cacao- and passion fruit-based desserts, food neophobia (assessed using a short standardized scale), and basic sociodemographic variables. The questionnaire consisted of a limited number of structured items (Likert-type and categorical), designed to minimize respondent fatigue, and was completed immediately before the sensory evaluation. The questionnaire was administered in the same controlled environment as the sensory evaluation. Food neophobia was treated as the most theoretically relevant individual-difference variable because, unlike age, which was used primarily to characterize the sample, and product familiarity, which was considered a situational control, it reflects a relatively stable food-related disposition that could plausibly moderate responses to extrinsic sensory cues across tasting conditions. For this reason, it was prioritized as the focal moderator when examining whether plate-color effects varied across participants.

Participants then received instructions on using the visual analog scales and the hedonic scale. They were informed that the session consisted of a sensory evaluation of dessert products and were asked to rate perceived taste attributes using standardized scales. They were not informed of the specific research objective regarding the effect of plate color on sensory perception, in order to minimize expectation bias and demand characteristics. Although the servings were presented using randomized alphanumeric codes under a double-blind protocol, the visual and sensory characteristics of the desserts may have allowed partial recognition of product identity during evaluation. The servings were presented sequentially following a balanced Latin square randomization scheme designed for four treatments (T1–T4) to counterbalance potential order and period effects.

The study procedure was organized into four sequential stages: (1) participant screening and eligibility verification, (2) administration of a pre-session questionnaire, (3) sensory evaluation of the experimental conditions under controlled settings, and (4) immediate recording of responses after each tasting.

Treatments were defined as crossed combinations of product type and plate color: T1 = Cacao-based dessert on a black plate; T2 = Cacao-based dessert on a red plate; T3 = Passion fruit-based dessert on a black plate; and T4 = Passion fruit-based dessert on a red plate. The balanced Latin square used to determine the presentation order is shown in Table 1, and the assignment of sequences to participants is presented in Table 2. Participants were randomly assigned to the presentation sequences, with approximately equal numbers allocated to each group to preserve the balance of the Latin square design. After tasting each serving, participants immediately recorded their evaluations using a digital form administered through Google Forms.

The independent variables were plate color (two levels: red and black) and product type (cacao-based dessert vs. passion fruit-based dessert). The focal dependent variables reported in this article were perceived sweetness and bitterness, measured using 100-point visual analog scales. Additional measures—sourness (100-point visual analog scale), overall liking (nine-point hedonic scale), and consumption intention (Likert-type scale)—were collected for complementary purposes but were not included as primary outcomes in the present analysis. Hunger level, product familiarity, and food neophobia were included as covariates in the analyses, whereas age was used primarily for sample characterization rather than as a focal explanatory variable, given the within-subject design and the absence of a specific age-based hypothesis.

Data analysis followed a structured sequence consistent with the study’s repeated-measures design. First, descriptive statistics were used to summarize the distribution of the variables. Second, inferential analyses were conducted according to the measurement scale of the outcomes. Continuous sensory ratings were analyzed using linear mixed-effects models, whereas ordinal outcomes were analyzed using cumulative link mixed models. When significant effects were detected, post hoc comparisons were performed with appropriate adjustments for multiple testing. For continuous sensory intensity ratings, linear mixed-effects models with a random intercept for participants were specified. The general form of the model was:

Yij = β0 + β1Colorij + β2Productij + β3(Color × Product)ij + Zijγ + ui + εij

where Yij represents the sensory response of participant i under condition j; β0 is the fixed intercept; β1, β2, and β3 correspond to the fixed effects of plate color, product type, and their interaction; Zijγ denotes the vector of covariates (hunger level, familiarity, and food neophobia); ui is the participant-specific random effect, assumed ui~N(0, σu2); and εij is the residual error term, with εij~N(0, σ2). Presentation order and evaluation period were additionally included as fixed effects to control for potential learning, fatigue, or carryover effects, and their statistical significance was assessed to verify the adequacy of the counterbalancing scheme. The mixed-effects framework explicitly accounts for the hierarchical structure of the data, where repeated evaluations are nested within participants.

Ordinal outcomes—specifically, overall liking and consumption intention—were analyzed using cumulative link mixed models. When significant main effects or interactions were detected, post hoc pairwise comparisons between plate color levels were conducted, with adjustments for multiple testing using Holm’s procedure or false discovery rate control, as appropriate. Model assumptions were assessed through graphical inspection of residuals, analysis of standardized residuals, and evaluation of influential observations. For ordinal models, the proportional odds assumption was examined. The significance level was set at α = 0.05. All tests were two-sided, and exact *p*-values were reported along with 95% confidence intervals and appropriate effect size measures.

Data processing and statistical analyses were performed using R (version 4.3.2), employing specialized packages for mixed-effects modeling and sensory analysis, including lme4, lmerTest, emmeans, and ordinal. The hypotheses, primary outcomes, and analysis plan were preregistered prior to data collection. Anonymized data and analysis scripts will be made publicly available in accordance with FAIR principles to support transparency and reproducibility. This analytical approach ensures that both within-subject dependencies and potential confounding effects are appropriately controlled.

## 3. Results

Linear mixed-effects models were used to evaluate the effects of plate color, product type, and their interaction on sensory perception, while accounting for repeated measurements and individual-level variability. 

Table 3 summarizes the hypothesis tests. The results provide strong evidence of a significant plate color × product interaction for both sweetness and bitterness, supporting the core hypothesis that the effect of visual context depends on the product’s sensory profile. Both directional hypotheses were supported, whereas no statistically significant evidence was found for moderation by food neophobia.

Taken together, these findings indicate that plate color does not exert a uniform influence on sensory perception across products, but instead selectively modulates dominant taste attributes in a product-specific manner.

### 3.1. Effects of Plate Color on Perceived Sweetness

The Type III ANOVA for perceived sweetness (Table 4) revealed significant main effects of plate color and product type, as well as a significant plate color × product interaction, indicating that the effect of plate color depended on the product evaluated.

Model estimates (Table 5) showed that the passion fruit-based dessert was perceived as sweeter than the cacao-based dessert. Importantly, this difference was amplified when the passion fruit-based dessert was served on a red plate, as reflected by the positive and statistically significant interaction term. In contrast, plate color had a comparatively minor effect on perceived sweetness in the cacao-based dessert.

**Figure 1:**
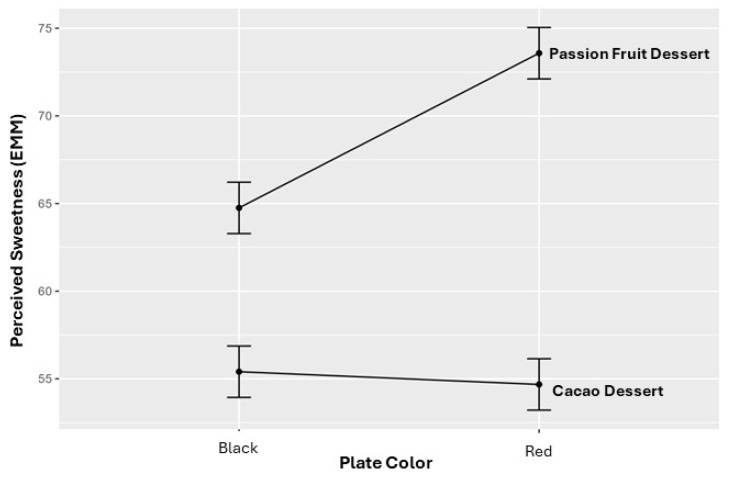
Estimated marginal means of perceived sweetness by plate color and product type. Values are expressed on a 0–100 visual analog scale (VAS) for cacao- and passion fruit-based desserts. Error bars represent 95% confidence intervals derived from the linear mixed-effects model.

This interaction pattern is illustrated in Figure 1, which shows that sweetness ratings increased markedly for the passion fruit-based dessert when served on a red plate, whereas plate color had a comparatively smaller effect on the cacao-based dessert. Together, these results indicate that plate color selectively enhances sweetness perception in products whose sensory profile is dominated by sweet–acidic attributes.

**Figure 2:**
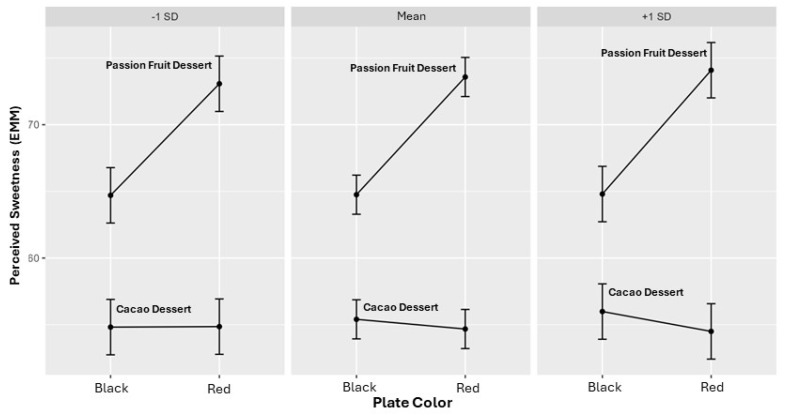
Predicted sweetness by food neophobia, plate color, and product type. Predicted values are expressed on a 0–100 visual analog scale (VAS) for cacao- and passion fruit-based desserts served on red and black plates.

Food neophobia did not exhibit a significant main effect on perceived sweetness, nor did it interact significantly with plate color or product type (Table 4). As shown in Figure 2, sweetness ratings followed parallel trends across neophobia levels, indicating no meaningful moderation of plate color effects.

The model demonstrated good explanatory power, with a marginal R^2^ of 0.52 and a conditional R^2^ of 0.62, indicating that a substantial proportion of variance was explained by fixed effects, with additional variability attributable to interindividual differences (ICC = 0.21). Diagnostic tests did not reveal violations of model assumptions (Table 6).

Directed contrasts (Table 7) confirmed that sweetness ratings for the passion fruit-based dessert were significantly higher on red plates than on black plates, supporting hypothesis H2b.

### 3.2. Effects of Plate Color on Perceived Bitterness

The Type III ANOVA for perceived bitterness (Table 8) revealed significant main effects of plate color and product type, as well as a significant plate color × product interaction, indicating that the effect of plate color depended on the product evaluated.

Model estimates (Table 9) indicated that the cacao-based dessert was perceived as markedly more bitter than the passion fruit-based dessert. Within the cacao-based dessert, serving the product on a black plate resulted in higher perceived bitterness relative to a red plate, whereas this contrast was attenuated for the passion fruit-based dessert.

**Figure 3:**
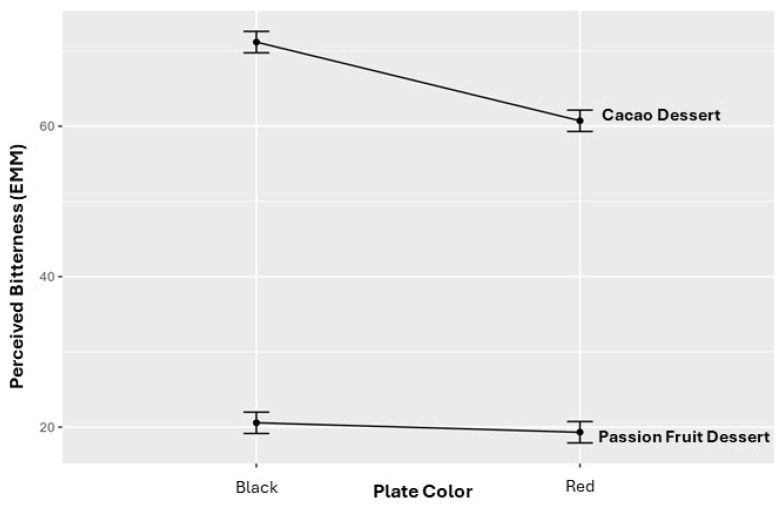
Estimated marginal means of perceived bitterness by plate color and product type. Values are expressed on a 0–100 visual analog scale (VAS) for cacao- and passion fruit-based desserts. Error bars represent 95% confidence intervals.

This interaction is visualized in Figure 3, which shows higher bitterness ratings for the cacao-based dessert when presented on a black plate, whereas plate color effects were attenuated for the passion fruit-based dessert.

**Figure 4:**
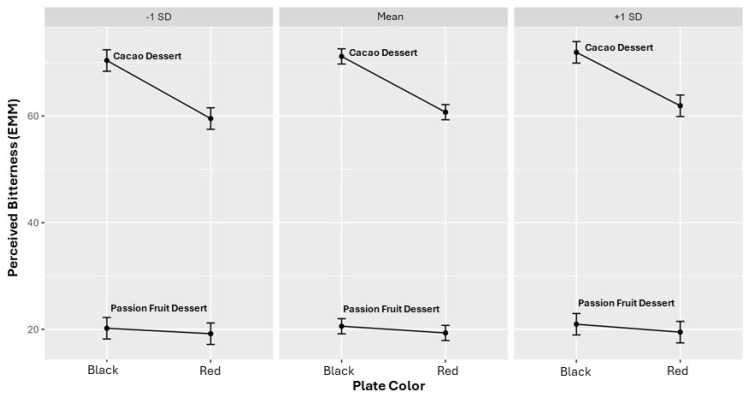
Predicted bitterness by food neophobia, plate color, and product type. Predicted values are expressed on a 0–100 visual analog scale (VAS) for cacao- and passion fruit-based desserts served on red and black plates.

Food neophobia did not exhibit significant main effects or interactions with plate color or product type (Table 9). As shown in Figure 4, predicted bitterness ratings followed parallel trends across levels of neophobia, indicating no meaningful moderation.

Directed contrasts (Table 10) confirmed that bitterness ratings for the cacao-based dessert were significantly higher on black plates than on red plates, supporting hypothesis H2a.

The bitterness model exhibited strong explanatory power (marginal R^2^ = 0.91; conditional R^2^ = 0.93), with diagnostic tests supporting the adequacy of model assumptions. Taken together, these results were consistent with the product-dependent pattern observed for sweetness, while showing no evidence of meaningful moderation by food neophobia.

## 4. Discussion

The present study provides empirical evidence that plate color modulates the sensory perception of traditional Ecuadorian desserts, and that this effect is not uniform across products but depends on the food’s intrinsic sensory profile. Specifically, serving passion fruit mousse on a red plate increased perceived sweetness, whereas presenting a cacao-based dessert on a black plate intensified perceived bitterness. These findings support the proposed hypotheses and reinforce the view that visual cues in the consumption context can actively shape gustatory experience rather than functioning as neutral background elements.

From a theoretical perspective, the results are consistent with the framework of crossmodal correspondences, which posits systematic associations between visual attributes, such as color, and specific taste qualities. Previous research has documented associations between reddish hues and sweetness, as well as between dark colors and bitterness, even in the absence of physicochemical changes in the food itself [4,5,6,8,9,10,11]. By replicating these patterns in traditional Ecuadorian products, the present study extends the empirical scope of crossmodal correspondence models and suggests that these associations may generalize across different cultural food contexts.

The significant interaction between plate color and product type observed for both sweetness and bitterness indicates that the influence of visual context is contingent on the dominant sensory attributes of the food. In the case of passion fruit, a product characterized by pronounced acidity, the red plate likely activated expectations of sweetness, resulting in perceptual assimilation that amplified perceived sweetness. This interpretation is consistent with expectation assimilation theory, which predicts that when contextual cues are congruent with intrinsic sensory properties, perceptual judgments shift toward the induced expectation [13,15,26,27].

Conversely, the increase in perceived bitterness for the cacao-based dessert served on a black plate is consistent with prior evidence linking dark colors with intense sensory attributes, including bitterness and flavor strength [2,7]. In products where bitterness constitutes a salient component of the sensory profile, plate color appears to function as an amplifying cue rather than as a compensatory signal. This suggests that the effects of color are shaped not only by color–taste correspondences but also by the relative salience of specific taste attributes within the product.

An important finding of the present study is the absence of evidence supporting moderation by food neophobia. Food neophobia was prioritized as the focal individual-difference variable because it represents a relatively stable food-related disposition that could plausibly influence expectation-based sensory judgments across tasting contexts, whereas age was treated primarily as a descriptive characteristic and product familiarity as a situational control. Although previous research has suggested that individual traits may influence sensitivity to contextual cues [28,29,30], the current results indicate that, under the experimental conditions employed, plate color effects were relatively homogeneous across participants. This finding aligns with the view that many crossmodal correspondences operate at an automatic, largely preattentive level, exerting their influence independently of stable personality traits [1,3,31,32,33].

From a methodological standpoint, the use of a randomized within-subject crossover design combined with linear mixed-effects modeling enabled the detection of interaction effects that might have remained undetected in between-subject designs. This approach responds to recent calls in sensory and consumer research for more sensitive experimental designs and analytically rigorous methods when studying contextual effects [17,18,26]. The convergence of results across global tests and post hoc contrasts further supports the robustness of the findings.

Beyond theoretical contributions, the findings have practical implications for gastronomy, culinary experience design, and food marketing. Plate color represents a low-cost, non-invasive tool for modulating sensory perception, particularly in products where balancing sweetness, acidity, and bitterness is desirable without altering formulation. In the Ecuadorian context, these results provide empirical support for the sensory positioning of culturally relevant products, such as cacao- and passion-fruit-based desserts, with potential applications in both local and international markets.

Finally, although the present study provides evidence for plate color effects under controlled conditions, future research could extend this work by incorporating additional contextual variables, such as plate material, ambient lighting, or more ecologically valid consumption environments. Examining culturally diverse samples would also help assess the generalizability of these effects and their interactions with local gastronomic norms.

### 4.1. Managerial Implications

The findings of this study provide relevant managerial implications for actors across the gastronomic and food value chain, particularly in contexts where sensory differentiation and consumer experience contribute to competitive advantage.

The results indicate that plate color can modulate key sensory attributes, such as sweetness and bitterness, without altering product formulation. From a managerial perspective, this suggests that presentation design can be considered a functional component of the consumption experience rather than a purely esthetic choice. Restaurants, cafés, and catering services may enhance perceived sensory attributes by aligning plate color with the dominant taste profile of the food served.

The observed pattern—where red plates enhance perceived sweetness in acidic desserts and black plates intensify bitterness in cacao-based desserts—suggests that visual presentation can be used to reinforce expected sensory characteristics. Such alignment may contribute to more consistent consumer evaluations and an improved overall consumption experience. Beyond tableware selection in on-premise consumption settings, these findings may also inform packaging, plated product presentation, and visual design strategies aimed at shaping sensory expectations before tasting.

The use of plate color as a sensory modulator also has potential implications for product reformulation and cost management. In contexts where reducing ingredients such as sugar is desirable, visual cues may serve as a complementary mechanism to support perceived sweetness or intensity. This may be particularly relevant in settings with cost constraints or regulatory pressures.

For traditional products, such as Ecuadorian cacao- and passion fruit-based desserts, the results offer practical insights for strengthening product presentation and differentiation. Plate color selection may be used to highlight characteristic sensory attributes, contributing to a more coherent and distinctive consumption experience, particularly in tourism-oriented or export markets.

From an experiential marketing perspective, the findings highlight the role of visual design elements in shaping product perception. Integrating plate color with broader elements of service design may support the creation of more consistent multisensory experiences.

The absence of significant moderation by food neophobia suggests that the observed effects are relatively stable across consumers. This may facilitate implementation, as strategies based on plate color do not appear to require fine-grained segmentation.

Finally, the study underscores the value of incorporating empirical evidence into decisions related to product presentation and service design. Experimental approaches and sensory evaluation methods can support more informed and systematic management of consumer experience.

### 4.2. Limitations and Directions for Future Research

Despite the methodological rigor of the experimental design and analytical approach, several limitations should be considered when interpreting the findings of this study.

The experiment was conducted in a controlled sensory laboratory, with standardized lighting, temperature, and evaluation conditions. While this strengthens internal validity and enables the isolation of plate color effects, it may limit ecological validity. In real-world consumption settings—such as restaurants or domestic environments—multiple contextual stimuli coexist, including variable ambient lighting, noise, social interaction, and menu presentation, which may interact with or attenuate the effects observed under controlled conditions.

The scope of the study is also constrained by product selection. Although two desserts with contrasting sensory profiles (cacao- and passion fruit-based desserts) were included, the range of food categories examined remains limited. Plate color effects may differ in other contexts, such as savory foods, beverages, or products with more complex aromatic or visual characteristics, thereby limiting the generalizability of the findings.

Similarly, the selection of plate colors was restricted to red and black, based on prior research on color–taste crossmodal correspondences. However, the study did not examine a broader range of hues nor systematically manipulate color dimensions such as saturation or brightness. These attributes may play a critical role in shaping sensory expectations and perceptual responses, warranting further investigation.

At the individual level, food neophobia was the only psychological trait considered as a potential moderator. Although no significant moderation effects were observed, other individual differences—such as sensory sensitivity, cultural background, or prior gastronomic experience—may influence responsiveness to visual contextual cues.

From a procedural standpoint, the within-subject design raises the possibility that participants may have formed implicit expectations during repeated evaluations. Although the study was conducted under a double-blind protocol and participants were not informed of the specific research objective, it cannot be entirely ruled out that some individuals became aware of contextual differences across conditions.

In addition, several limitations relate to the characterization of the visual stimuli. The colors of the plates and desserts were not instrumentally measured in CIELAB coordinates, and no photographic record of the plated samples was collected. Likewise, the relative visual proportion between the dessert and the plate surface was not quantitatively assessed. Although portion size and plating position were standardized, the absence of objective colorimetric and spatial measurements limits the precision with which the visual conditions can be replicated and analyzed.

A further limitation concerns the interpretation of sensory intensity measures obtained through visual analog scales (VAS). While these instruments are widely used and validated in sensory research, they capture participants’ reported quantification of perceived intensity rather than directly measuring the underlying perceptual experience. Consequently, the present study cannot fully disentangle whether plate color influences perceptual experience itself or the cognitive process by which that experience is translated into scale-based responses.

Finally, although the study relied on well-established subjective measures, it did not incorporate physiological or neurocognitive indicators that could provide deeper insight into the mechanisms underlying crossmodal correspondences.

Building on these limitations, several directions for future research emerge. Replication of the present study in real or semi-naturalistic consumption environments—such as restaurants or immersive dining settings—would allow assessment of the robustness of plate color effects under more complex conditions. Expanding the range of food products and systematically manipulating color properties (e.g., hue, saturation, brightness, and contrast) would further refine the understanding of visual–taste interactions. Future studies should also consider additional individual-level variables and incorporate multimodal approaches that combine subjective evaluations with physiological or neurocognitive measures to better elucidate the underlying perceptual and cognitive processes.

## 5. Conclusions

This study provides empirical evidence that plate color functions as a relevant contextual cue in sensory evaluation, influencing participants’ reported intensity ratings of key taste attributes in traditional Ecuadorian desserts without altering their physicochemical properties. The findings support the view that flavor evaluation emerges from a multisensory process in which visual cues contribute systematically to how sensory experiences are interpreted and quantified.

The results further indicate that the effect of plate color is not uniform, but depends on the dominant sensory profile of the product. In desserts characterized by pronounced acidity, such as passion fruit-based preparations, warm plate colors were associated with higher reported sweetness, whereas in products where bitterness is a salient attribute, such as cacao-based desserts, darker plate colors were associated with higher reported bitterness. These findings suggest that visual cues interact with intrinsic product characteristics in a context-dependent manner, rather than producing generalized effects.

From a theoretical perspective, the study extends existing evidence on color–taste crossmodal correspondences to traditional Latin American food products, contributing to the external validity of multisensory perception frameworks. The absence of significant moderation effects of food neophobia suggests that these associations may operate at a broadly shared perceptual–cognitive level, although further research is needed to explore other individual-level moderators.

Methodologically, the combination of a randomized within-subject crossover design and linear mixed-effects modeling proved suitable for detecting contextual interaction effects of moderate magnitude, providing a replicable framework for future research in sensory and consumer science.

From a practical standpoint, the findings indicate that visual presentation elements such as tableware color can influence how consumers report and evaluate sensory attributes, which may be relevant for gastronomy and food marketing strategies. However, these implications should be interpreted with caution, given that the study does not allow definitive conclusions about whether visual context alters perceptual experience itself or its cognitive quantification.

Overall, the study contributes to a more nuanced understanding of the role of visual context in sensory evaluation and highlights the need for further research integrating behavioral, perceptual, and physiological approaches to disentangle underlying mechanisms.

## Figures and Tables

**Table 1:** Balanced Latin square design for plate color presentation.

Sequence	Period 1	Period 2	Period 3	Period 4
A	T1	T2	T3	T4
B	T2	T3	T4	T1
C	T3	T4	T1	T2
D	T4	T1	T2	T3

**Table 2:** Assignment of presentation sequences to participants.

Participant Group	Assigned Sequence
Group 1 (*n* = 26)	A
Group 2 (*n* = 27)	B
Group 3 (*n* = 26)	C
Group 4 (*n* = 26)	D

**Table 3:** Hypothesis tests and directed contrasts estimated using linear mixed-effects models.

Hypothesis	Contrast Description	Dependent Variable	Statistical Evidence	*p*-Value
H1	Plate color × product interaction	Sweetness	F(1, 291) = 51.63	<0.001
	Bitterness	F(1, 291) = 48.57	<0.001	
H2a	Cacao-based dessert: black plate > red plate	Bitterness	Δ = 10.46 (VAS)	<0.001
H2b	Passion fruit-based dessert: red plate > black plate	Sweetness	Δ = 8.83 (VAS)	<0.001
H3	Plate color × product × food neophobia interaction	Sweetness	F(1, 291) = 0.84	0.361
	Bitterness	F(1, 291) = 0.25	0.619	

Note. Contrasts were estimated using linear mixed-effects models with a random intercept for participant. Directed contrasts (H2a and H2b) were evaluated using post hoc comparisons based on estimated marginal means (emmeans). Δ values represent mean differences on visual analog scales (VAS; 0–100). H2a and H2b represent directed post hoc contrasts specified a priori to operationalize the directional hypothesis H2.

**Table 4:** Type III ANOVA of fixed effects for perceived sweetness (VAS 0–100).

Effect	Sum of Squares	df (num)	df (den)	F	*p*-Value
Plate color	1640.09	1	291	37.12	<0.001
Product type	19,944.84	1	291	451.35	<0.001
Food neophobia	11.15	1	98	0.25	0.617
Period	58.60	3	291	0.44	0.723
Plate color × Product	2281.42	1	291	51.63	<0.001
Plate color × Neophobia	2.30	1	291	0.05	0.820
Product × Neophobia	0.56	1	291	0.01	0.910
Plate color × Product × Neophobia	36.98	1	291	0.84	0.361

Note. Type III ANOVA derived from a linear mixed-effects model with a random intercept for participant. Sweetness was measured using a visual analog scale (0–100). The plate color × product interaction corresponds to the test of hypothesis H1.

**Table 5:** Linear mixed-effects model estimates for perceived sweetness.

Term	β (Estimate)	Standard Error	df	t	*p*-Value
Intercept (cacao-based dessert, black plate)	55.15	0.95	388.97	58.35	<0.001
Plate (Red vs. Black)	−0.73	0.94	291	−0.77	0.440
Product (passion fruit-based dessert vs. cacao-based dessert)	9.35	0.94	291	9.94	<0.001
Food neophobia	0.58	0.75	345.28	0.78	0.437
Period 2	0.41	0.94	291	0.43	0.665
Period 3	−0.19	0.94	291	−0.20	0.842
Period 4	0.80	0.94	291	0.85	0.394
Red plate × Passion fruit-based dessert	9.55	1.33	291	7.19	<0.001
Red plate × Neophobia	−0.76	0.94	291	−0.81	0.419
Passion fruit-based dessert × Neophobia	−0.54	0.95	291	−0.57	0.571
Red plate × Passion fruit-based dessert × Neophobia	1.22	1.34	291	0.91	0.361

Note. Coefficients were estimated using a linear mixed-effects model with a random intercept for participant. The reference level corresponds to the cacao-based dessert served on a black plate. The highlighted coefficient (red plate × passion fruit-based dessert) supports the directional hypothesis H2b.

**Table 6:** Model fit metrics and diagnostics for the linear mixed-effects model of perceived sweetness.

Metric	Value
Marginal R^2^	0.52
Conditional R^2^	0.62
Intraclass correlation coefficient (ICC)	0.21
DHARMa test	*p*-value
Residual uniformity	0.627
Dispersion	0.760
Outliers	0.676

**Table 7:** Directed contrasts (emmeans) for perceived sweetness.

Hypothesis	Product	Contrast	Δ (Mean Difference)	Standard Error	df	t	*p*-Value
H2b	Passion fruit-based dessert	Red plate > Black plate	8.8262	0.9401	291	9.3887	<0.001

Note. Mean differences (Δ) correspond to directed contrasts between plate colors within each product, estimated from adjusted marginal means. Positive values indicate higher perceived sweetness on red plates relative to black plates. Contrasts were evaluated under a linear mixed-effects model with a random intercept for participant.

**Table 8:** Type III ANOVA of fixed effects for perceived bitterness.

Effect	Sum of Squares	df (num)	df (den)	F	*p*-Value
Plate color	3429.57	1	291	78.70	<0.001
Product type	211,630.49	1	291	4856.30	<0.001
Food neophobia	84.32	1	98	1.93	0.167
Period	39.70	3	291	0.30	0.823
Plate color × Product	2116.65	1	291	48.57	<0.001
Plate color × Neophobia	0.99	1	291	0.02	0.880
Product × Neophobia	51.35	1	291	1.18	0.279
Plate color × Product × Neophobia	10.80	1	291	0.25	0.619

**Table 9:** Linear mixed-effects model estimates for perceived bitterness.

Term	β (Estimate)	Standard Error	df	t	*p*-Value
Intercept (cacao-based dessert, black plate)	71.36	0.92	388.30	77.15	<0.001
Plate (Red vs. Black)	−10.46	0.93	291	−11.20	<0.001
Product (passion fruit-based dessert vs. cacao-based dessert)	−50.60	0.93	291	−54.20	<0.001
Food neophobia	0.77	0.73	358.85	1.05	0.292
Period 2	0.11	0.94	291	0.12	0.906
Period 3	−0.15	0.94	291	−0.16	0.877
Period 4	−0.71	0.94	291	−0.76	0.448
Red plate × Passion fruit-based dessert	9.20	1.32	291	6.97	<0.001
Red plate × Neophobia	0.43	0.94	291	0.46	0.647
Passion fruit-based dessert × Neophobia	−0.39	0.94	291	−0.42	0.678
Red plate × Passion fruit-based dessert × Neophobia	−0.66	1.33	291	−0.50	0.619

**Table 10:** Directed contrasts (emmeans) for perceived bitterness.

Hypothesis	Product	Contrast	Δ (Mean Difference)	Standard Error	df	t	*p*-Value
H2a	Cacao-based dessert	Black plate > Red plate	10.4569	0.9336	291	11.2009	<0.001

Note. Mean differences (Δ) correspond to directed contrasts between plate colors within each product, estimated from adjusted marginal means. Positive values indicate higher perceived bitterness on black plates relative to red plates. Contrasts were evaluated under a linear mixed-effects model with a random intercept for participant.

## Data Availability

The original contributions presented in this study are included in the article. Further inquiries can be directed to the corresponding author.
